# Ontology-Based Graphs of Research Communities: A Tool for Understanding Threat Reduction Networks

**DOI:** 10.3389/frma.2020.00003

**Published:** 2020-06-09

**Authors:** John Ambrosiano, Benjamin Sims, Andrew W. Bartlow, William Rosenberger, Mark Ressler, Jeanne M. Fair

**Affiliations:** ^1^Information Systems and Modeling, Los Alamos National Laboratory, Los Alamos, NM, United States; ^2^Statistical Sciences, Los Alamos National Laboratory, Los Alamos, NM, United States; ^3^Biosecurity and Public Health, Los Alamos National Laboratory, Los Alamos, NM, United States

**Keywords:** collaboration, connectional intelligence, bioinformatics, global health, ontology, social network, threat reduction network

## Abstract

Scientific research communities can be represented as heterogeneous or multidimensional networks encompassing multiple types of entities and relationships. These networks might include researchers, institutions, meetings, and publications, connected by relationships like authorship, employment, and attendance. We describe a method for efficiently and flexibly capturing, storing, and extracting information from multidimensional scientific networks using a graph database. The database structure is based on an ontology that captures allowable types of entities and relationships. This allows us to construct a variety of projections of the underlying multidimensional graph through database queries to answer specific research questions. We demonstrate this process through a study of the U.S. Biological Threat Reduction Program (BTRP), which seeks to develop Threat Reduction Networks to build and strengthen a sustainable international community of biosecurity, biosafety, and biosurveillance experts to address shared biological threat reduction challenges. Networks like these create connectional intelligence among researchers and institutions around the world, and are central to the concept of cooperative threat reduction. Our analysis focuses on a series of seven BTRP genome sequencing training workshops, showing how they created a growing network of participants and countries over time, which is also reflected in coauthorship relationships among attendees. By capturing concept and relationship hierarchies, our ontology-based approach allows us to pose general or specific questions about networks within the same framework. This approach can be applied to other research communities or multidimensional social networks to capture, analyze, and visualize different types of interactions and how they change over time.

## Introduction

Since the 1990s, Cooperative Threat Reduction (CTR) programs of the United States and other countries have been implemented as high return-on-investment approaches for reducing the threat of infectious diseases and epidemics at modest cost (Smithson, 2016). These programs seek to build international networks of infectious disease laboratories, professionals, and scientists in order to increase the world's ability to detect and diagnose infectious disease outbreaks. In this paper, we focus on the application and impact of the Biological Threat Reduction Program (BTRP), a CTR program within the Defense Threat Reduction Agency (DTRA) of the U.S. Department of Defense. This program seeks to build research communities which it calls Threat Reduction Networks (TRNs).

Programs like BTRP need ways of quantifying and visualizing the impact of their network-building efforts. While bibliometric and other approaches using public data can be helpful in this regard, many scientific research networks come together around a much wider array of collaborations and interactions, including meeting and workshop attendance; interactions within and among institutions, departments, and regions; collaborations on projects and proposals; participation in informal networks of like-minded specialists; and ongoing relationships with sponsors and funding agencies.

In order to capture these diverse aspects of research networks like TRNs, it is helpful to describe them as networks that include multiple types of nodes and multiple types of relationships, which are sometimes called multidimensional or heterogeneous networks (Contractor et al., 2011; terminology is discussed in more detail below). Based on data provided to us by BTRP, we were able to construct such a network, where types of nodes include projects, meetings, people, organizations, groups, documents, and research topics; and possible relationships include authorship, affiliation, attendance, and residence, among others.

Working with multidimensional graphs of this sort is challenging because they can quickly become very complex, creating problems for visualization and analysis. To cope with this complexity, it is helpful to be able to sort, filter, and project elements of the resulting graph so that particular aspects of the network can be extracted for analysis and visualization, while maintaining the integrity of the entire underlying graph data. The main contribution of this paper is to demonstrate a method for storing, manipulating, visualizing, and analyzing multidimensional graphs using an ontology-based graph database. To do this, we created an ontology, SCINET, that includes the necessary entities and relationships to describe a multidimensional scientific research network; encoded BTRP source documents using this ontology structure; stored the resulting data in a graph database; and used database queries to construct appropriate subgraphs for visualization and analysis.

The main goal of this paper is to describe the development and use of our ontology-based graph database approach in sufficient detail so that others may adapt and extend this approach to their needs in studying similar networks. To do this, we provide some context on TRNs and scientific networks in general; review related research in ontologies and multidimensional networks; describe our ontology and database development process in detail; and finally provide a basic example of how this approach can be used to study one particular aspect of a TRN, namely how attendance at a series of workshops built up a network of researchers over time. We hope that this will provide a useful overview of our approach, as well as some insights on TRNs that may be of interest to the CTR community.

## Background and Related Research

### Importance of Scientific Collaboration for Reducing Infectious Disease Threats

Science is an increasingly collaborative enterprise. Studies have shown a consistent increase in collaboration across multiple fields over the past 30–40 years, as indicated by the average number of authors per publication and numerous other indicators (Sonnenwald, 2007; Leahey, 2016; Wagner et al., 2017). Collaborations are also increasingly international, in part due to the diminishing role of proximity as a prerequisite for collaboration, driven by the increasing prevalence of air travel as well as communication technology (Leydesdorff and Wagner, 2008; Hoekman et al., 2010; Storme et al., 2017). Collaboration has many benefits in terms of solving more complex problems, overcoming increasing scientific specialization, sharing resources, producing more impactful results, and in general facilitating the production and dissemination of knowledge (Sonnenwald, 2007; Bozeman et al., 2013; Leahey, 2016). Collaborations also help developing countries build capacity and connections to the international scientific community (Owusu-Nimo and Boshoff, 2017). This growth in collaboration creates an increasingly large and well-connected scientific network across the globe (Leydesdorff et al., 2013). While coauthorship data provide documentation of this increasingly connected network, connectivity is driven by many other activities, including both formal and informal meetings as well as conferences and workshops that bring dispersed research communities together to meet face-to-face (Sonnenwald, 2007; Storme et al., 2017).

Given the benefits of collaboration, governmental agencies and organizations actively seek to foster and strengthen science networks, including those involved in global health, where investments in network-building activities are seen as efficient and effective measures for increasing research capacity (Nelson et al., 2018). Global health programs are (1) establishing strong international collaborative research networks (Fair et al., 2016), (2) building capacity for research in partner countries through training (Johnson et al., 2015), and (3) increasing networks of support for biosafety and biosecurity across regions and among countries that support the International Health Regulations (Standley et al., 2015). Measuring the return on investment for scientific networks, in particular TRNs, is a critical need for sponsoring agencies to show the value of these networks for reducing the threat of infectious diseases globally (Fair et al., 2016).

Emerging infectious diseases (EIDs) are a significant threat to global health, with ~75% of these caused by zoonotic pathogens (Taylor et al., 2001; Woolhouse and Gowtage-Sequeria, 2005). With the complexity of multiple hosts and vectors in a rapidly changing environment, there is an increased need for all countries to use the best science and technology for diagnosis, detection, and reporting on EIDs, especially those involving dangerous pathogens. In 2020, the world is experiencing first hand a zoonotic infectious disease pandemic caused by a coronavirus that most likely emerged from bats (Andersen et al., 2020). The program of BTRP and their created networks of scientists were developed specifically to reduce the threat of infectious diseases. Achieving the goal of increasing disease detection, diagnostics, and reporting capabilities worldwide requires countries with highly developed capabilities in these areas to share their expertise and create training opportunities for new infectious disease professionals in the latest advancements in biosurveillance. In a previous study on the outcomes from a CTR training, a high return on investment was demonstrated for a single workshop with participants from several countries (Fair et al., 2016). The authors of the study focused on a 1-week training in bat surveillance methods that covered proper capture and handling of bats, sampling approaches, and the latest molecular techniques for identifying infectious pathogens such as coronaviruses. Not only did the participants get training in biosafety and the newest molecular techniques, but a small TRN emerged from the workshop, leading to several collaborations and partnerships that developed research proposals, standard operating procedures, and two scientific papers. This is just one example of how the active encouragement and development of TRNs can increase the spread of cutting-edge biosurveillance methods, as well as coordination among infectious disease researchers among countries to address the challenges of pathogens.

By strengthening connections and trust, scientific networks increase connectional intelligence among researchers around the world. As defined by Dhawan and Saj-Nicole (2015), connectional intelligence is “the capability to consistently deliver breakthrough innovation and results by harnessing the value of relationships and networks.” Threat reduction requires connectional intelligence that fosters innovation to solve highly complex challenges, quickly marshal resources and knowledge, enable interdisciplinary science breakthroughs, and develop future leaders globally to work together to reduce the threat of infectious diseases. Social network analysis approaches are essential tools for measuring connectional intelligence within scientific networks, including TRNs. Being able to track the return on investment of science networks or trainings is critical for understanding how best to sustain and support these networks.

### Threat Reduction Networks

The increasing complexity and irregularity of threats to national and global health security has led to new and novel countermeasures. Social networks can both contribute to and combat emerging threats facing the world. For example, terror networks have evolved to be highly connected globally, which increases their reach and potential threat (Sageman, 2004). On the other hand, emerging infectious diseases, which continue to increase globally (Jones et al., 2008; Rosenberg et al., 2018), have been shown to be successfully mitigated through collaborative social networks of infectious disease laboratories, professionals, and scientists (Albiger et al., 2018). Networks of scientists and laboratories are considered primary tools for increasing the capability to detect and diagnose infectious disease outbreaks.

The mission of BTRP is to reduce the threat posed by pathogens and diseases of security concern, related materials and expertise, and terrorist acquisition and use of biological weapons. To do this, BTRP works collaboratively with partner countries, international and non-governmental organizations, academia, and the U.S. interagency to strengthen biosecurity, biosafety, and disease surveillance competencies that enable partner countries to more effectively detect, diagnose, report, and contain disease outbreaks. Robust research networks are critical for preventing outbreaks, epidemics, and potential pandemics, as well as for combatting the nefarious use of biological agents. Within this context, BTRP created defined communities of scientists called TRNs to strengthen and build a sustainable community of biosecurity, biosafety, and biosurveillance capabilities and practitioners. TRNs provide a coordinating function among scientists, institutions, and regional partners to address shared biological threat reduction challenges. These networks are similar in construct to the U.S. National Science Foundation's Research Coordinated Networks, which are designed to bring scientists together around a topic or field. TRNs are typically built around a specific topic, such as bat-borne zoonotic infectious diseases, as a way to support meetings, collaborations, and relationship building. Another role of TRNs can be to develop training and strengthen capabilities in countries to create sustainable networks of scientists to support each other after initial funding ends. The motivation behind the creation of TRNs can be characterized by the phrase “if we wait until the first day of an outbreak to exchange business cards, the pathogen has already won.”

The primary objectives of research-focused TRNs, as defined by BTRP, are to (1) convene multi-disciplinary researchers, health implementers, policy makers, and funding authorities to identify and prioritize research needs and gaps; (2) characterize the distribution, prevalence, and ecology of infectious disease threats; (3) identify, evaluate, and implement sustainable consensus or “gold standard” assays and case definitions to determine if better standards are needed for detection in laboratory and clinical settings; and (4) increase awareness of infectious disease threats amongst at-risk populations, clinicians, laboratory staff, and national decision makers to encourage better surveillance, prevention, detection, and response. TRNs also serve as a forum for sharing of data, Standard Operating Procedures, and samples between researchers.

### Social Network Analysis

Social network analysis, particularly based on bibliometric data, is recognized as an important way of documenting the structure of scientific communities (Newman, 2001a,b, 2004; Barabási et al., 2002; Yan and Ding, 2009; Rafols et al., 2010; Abbasi et al., 2012; Yan and Guns, 2014; Li et al., 2017; Zhang et al., 2017, 2018).

In its most basic form, social network analysis focuses on networks composed of a single type of node, connected by a single type of link or edge, with edges often weighted by intensity. For example, in such a network, the nodes might be scientists, edges might indicate that two scientists have collaborated on at least one journal article, and each edge might be assigned a weight corresponding to the number of papers they co-authored. However, as larger and more complex network datasets have become available, researchers have begun to ask more sophisticated questions that cannot always be addressed in the context of a single dimension of interaction. For studying scientific collaboration, for example, we might want to include additional nodes like research institutes, countries, conferences, or objects of study; and we might want to use edges to capture additional types of relationships, for example that two journal articles refer to the same organism, or that two conferences are linked by a number of common participants (Contractor et al., 2011; Kas et al., 2012).

### Ontologies and Multidimensional Networks

For maximum flexibility, it can be helpful to represent these diverse relationships within a single graph consisting of multiple types of nodes and multiple types of edges, a *multidimensional network* using the terminology proposed by Contractor et al. (2011) (other terms like heterogenous networks or multimodal networks are sometimes used to describe this type of network, but may also be applied to graphs with only one type of node and multiple types of edges; the terminology used by Contractor et al. provides more precision). This kind of network can also be thought of as a form of *multilayer network*, because the types of nodes can be considered as different layers in the graph (Boccaletti et al., 2014; Kivelä et al., 2014; Mcgee et al., 2019).

**Figure 1:**
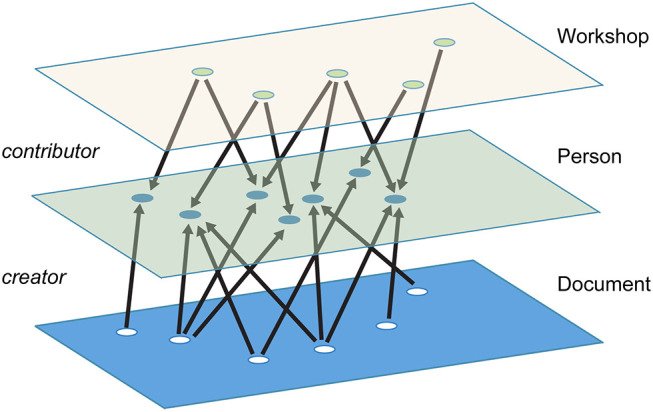
An example of a multidimensional, multipartite network with workshop, person, and document layers. Documents are connected to people by “creator” links, while workshops are connected to people by “contributor” links.

In this article, we map collaborations and interactions within the BTRP community as a multidimensional network. More specifically, our network is structured in the form of a multipartite graph, in which each type of node can have a direct connection only to nodes of a different type (Figure 1). For example, there cannot be a direct link between two people in our network; two people can only be linked if they have some other entity in common, like a paper they co-authored or a conference they both attended. The advantage of this structure is that it makes it possible to easily generate subgraphs of specific node and edge types relevant to a particular research question through a process of projection (Horvát and Zweig, 2013). For example, we might want to project the author-document network onto the authors, which would create a network of authors linked by edges that represent co-authorship, or onto the documents, which would create a network of documents linked by edges that indicate authors in common. Or we might want to go a step further and project an author-document-institution network onto institutions—for example to show how universities are connected via professors who co-author papers with professors at other universities.

This flexibility comes at a cost, however; the multiple node and edge types in a multidimensional, multipartite network can lead to complex graphs that are difficult to navigate, understand, and visualize. To manage this complexity, it is helpful to have a systematic way of categorizing types of nodes and edges and the possible ways they can connect with other types of nodes and edges. This requires a semantic model of their relationships, which can be captured in the form of an ontology, which in this context refers to a systematic way of categorizing and mapping the types of entities and relationships in a particular domain (Arp et al., 2015; Powell, 2015). As an example, a prominent ontology in the biomedical world is the Gene Ontology, which captures the possible relationships between cellular components, molecular functions, and biological processes, and provides an underlying structure for many biological databases (Ashburner et al., 2000; The Gene Ontology Consortium, 2018). Here, our domain of analysis is the bioscience research community, so the main entities we are concerned with are things like authors, publications, conferences, and research institutions—although to capture the community's research activity, we also include biological entities like hosts, pathogens, and diseases. We provide more specifics on this ontology below.

A number of studies have pointed out the advantages of ontology-based networks for social network analysis and visualization, in particular as a basis for selecting and visualizing only certain types of relationships within the network (Shen et al., 2006; Wu and Li, 2009; Chen et al., 2010; Ahmed et al., 2014; Boudebza et al., 2015). However, we are not aware of any previous effort to systematically implement such an ontology in the form of a graph database to analyze a particular research community, as we do here. Due to missing data and other limitations of our current BTRP dataset, in this paper we limit our focus to visual representation and descriptive analysis of a subset of BTRP-supported genomics and bioinformatics training participants. However, we believe our ontology-driven graph database approach provides a usable, scalable solution for storing, analyzing, and visualizing multidimensional networks that can be extended and automated for future analysis efforts.

## Materials and Methods

### Ontology and Database Development

Sci-Net is our term for the overall framework we have developed for analyzing scientific social networks. It comprises (1) an ontology that defines possible entities and relationships (which we call the SCINET ontology), (2) a collection of data describing the social network, and (3) a semantic database that implements the ontology structure and allows us to query the social network on multiple dimensions.

### Creating the Ontology

To develop an ontology for this study, we relied on information technology and standards developed over decades as part of the Semantic Web effort—a term coined by Tim Berners-Lee, the inventor of the World Wide Web, to brand an international collaboration to develop standards for semantically annotating content on the internet. The sophisticated knowledge modeling and management tools that emerged from the Semantic Web collaboration are collectively referred to as semantic technology. Semantic technology and standards allow ontologies to be shared, so that complex ontologies can be created from them. Information encoded using ontologies takes the form of a knowledge graph or semantic network (Sowa, 1992).

In developing the SCINET ontology, we leveraged a number of existing ontologies for key concepts and relationships. The Sci-Net dataset describes a network of social relationships involving agents such as organizations, groups, and people, and the activities (e.g., projects, workshops, meetings) in which they participate. It is the confluence of people and their affiliated institutions, and the activities in which they engage, that fosters collaborative work. Therefore, the key entities and relationships of an appropriate ontology must be able to describe both social networks and collaborative workflows. Since we are interested in research collaborations, our ontology must also incorporate ontologies that cover the relevant research topics.

With these requirements in mind, the SCINET ontology synthesizes the following ontologies and vocabularies:

- Dublin Core Metadata Terms (DC-Terms): Relationships for annotating documents and identifying their creators and subjects.
- Friend of a Friend (FOAF): Concepts identifying documents, persons, organizations, groups and group membership.
- The Bibliographic Ontology (BIBO): An ontology for describing creative works that extends existing ontologies for annotation such as DC-Terms.
- W3C Organization Ontology (ORG): A FOAF extension for describing formal organizations and their structure, a standard of the World Wide Web Consortium (W3C).
- NCBI Taxonomy (NCBI): A comprehensive controlled vocabulary for the classification of organisms used in Sci-Net to identify hosts, pathogens, and vectors in relationship to diseases as subjects of research collaborations.
- The Disease Ontology (DO): An ontology of diseases that references the International Classification of Diseases (ICD) standard, but is more suitable to annotating topics than the ICD.
- ISO 3166: An international, standard vocabulary for identifying geographic regions (e.g., countries) and subregions (e.g., states, provinces).
- The Abstract Process Ontology (APRO): A high-level ontology for describing process flows developed at Los Alamos and extended in Sci-Net to describe workflow activities, their participants, and the products that result from them.
- Time Ontology (TIME): An ontology of temporal concepts, a standard of the W3C, used to register the time and duration of events and activities in the SCINET ontology.

**Figure 2:**
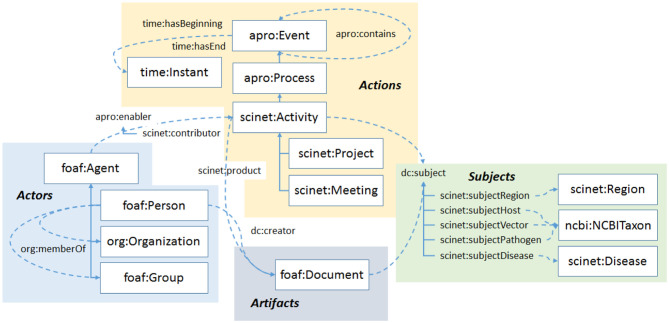
An outline of the SCINET ontology. The ontology brings together several types of entities familiar in workflow analysis such as actors, actions, and artifacts. Each of these can be linked to particular subjects.

SCINET customizes, extends, and combines all these ontologies to provide comprehensive coverage for the kind of network we describe in this paper. A high-level schematic of the main concepts and relationships in the ontology is shown in Figure 2. The main sections of the ontology are the shaded regions in the schematic. We see in the lower left of the schematic, in blue, the entities used in static social network analysis. Mainly adopted from the FOAF ontology and Dublin Core Terms, they involve various types of agents such as persons, organizations, and groups and their interrelations. In SCINET, agents are actors, i.e., their role is to enable actions, or in workflow terms, to participate in activities. The workflow portion of the ontology, the section shaded yellow in the upper middle portion of the diagram, shows that an activity is a kind of process, one in which human agents, either as individuals or in the aggregate, are the main enablers, and that a process is a kind of event, one during which a process unfolds. Projects and meetings are specific kinds of activities in the ontology. Although not shown, activities are elaborated further, with programs being a kind of activity that may involve one or more projects. Also, meetings can include specific kinds like workshops and conferences. Although it was not necessary for our current purposes, many of the static elements of the ontology, such as organizations or membership relationships, could also in principle have a temporal element, i.e., a beginning and an end.

Workflow activities produce artifacts. In research activities, the main class of artifacts is documents. Document artifacts (depicted at the bottom of Figure 2, shaded gray) are further elaborated as specific kinds such as journal articles, conference proceedings, reports, and so on. These classes are borrowed from the BIBO ontology.

The subject portion of the SCINET ontology is shaded green in the lower right of the schematic. Because the Sci-Net framework was developed to model research collaborations related to emerging global diseases, the subjects most relevant to the ontology are diseases, hosts, pathogens, and vectors, as well as the regions in which they may be found, or in which research projects were chosen to focus. It is worth noting that, except for specific subject areas, most of the concepts and relationships in SCINET would apply to any field of collaborative research. It is mainly the subject ontologies that would change when SCINET is extended to new research areas.

### Data Capture

For purposes of managing the data collection, Sci-Net data falls into one of two sub-collections:

- Supporting Data—the data that create the context for the research network. These include the NCBI taxonomy of organisms, the disease classification hierarchy, and countries and their subregions, as well as useful groupings of regions such as continents, and certain geopolitical groupings (e.g., the World Health Organization (WHO) regions). These data seldom change and rarely need to be updated once captured.
- Research Data—these are the data that comprise the networks of people, organizations, activities, events, and documents that are the main focus of interest in Sci-Net. These data change more frequently. Additions to the database are typically entities in this category.

For the supporting data, some ontologies and vocabularies were easily acquired from well-managed sources in a readily-imported form. Acquiring data from other sources required varying degrees of web scraping and data wrangling.

Research data was obtained primarily from reports and internal documentation provided to us by BTRP. These included annual reports and meeting and workshop reports, which contained research project abstracts; lists of journal publications and manuscripts authored by BTRP participants; and lists of workshop participants. In addition, we drew on a Frontiers Research Topics edited volume featuring 24 contributions from BTRP participants (Fair et al., 2017). We focused on these BTRP-specific data sources because they captured many projects and relationships that might not yet have translated into journal publications. In addition, since we did not have access to a complete list of BTRP participants and their affiliations, focusing on these sources gave us a way of limiting our scope to BTRP-relevant work.

The diverse and non-standardized nature of our research data sources created some challenges, however. Most significantly, it required us to manually encode much of the information in these documents as triples to populate the database. As we did this encoding, we also manually maintained canonical lists of people, organizations, and events, creating unique identifiers to facilitate disambiguation of these entities where their names had several variants. It would be desirable to automate these processes in the future. In addition, many of our data sources provided only partial snapshots of the BTRP network at particular moments in time, meaning that some types of information and some time periods were sampled in much more detail than others. This is one reason why our analysis in the present paper focuses on attendance at sequencing workshops, which was an aspect of the network that was very well-documented.

### Database Development

The kind of database best suited to the Sci-Net project is variously called a *semantic database*, a *graph database*, or a *triple store*. The database is semantic because queries on the database are guided by ontologies; it is a graph database because the set of all instances of concepts and relationships stored in the database forms a directed graph; and it is a triple-store because the data is logically equivalent to a collection of subject-predicate-object triples. It is the predicate-based logic of these triples that gives semantic databases superior power and flexibility over relational databases in analyzing semantic networks. For this effort, we used the GraphDB semantic graph database, which makes use of the Resource Description Framework (RDF) standard for representing triples (http://graphdb.ontotext.com).

Extracting data from a semantic database or triple-store involves forming queries based on the W3C standard for RDF data called SPARQL (pronounced “sparkle”) which stands for SPARQL Protocol and RDF Query Language. SPARQL queries are based on the fact that an ontology, along with the data organized and stored in relation to it, is a graph whose edges are logical facts, or subject-predicate-object triples, encoded in RDF or its equivalent. For example, the triples: (fair_jeanne, type, Person), (fair_jeanne, name, “Jeanne Fair”), and (fair_jeanne, alias, “Jeanne M. Fair”) describe one of the authors as belonging to the class Person and give her preferred name along with an alias.

SPARQL is a simple logical protocol where relevant triples are listed with variables standing in for the information desired. The database resolves the query by returning all the triples that satisfy the missing values of the variables. For example, the following query will return the identifiers for all triples whose subject is in the class Person, along with those objects that define the subject's name and any aliases associated with them:


select ?person ?alias where{
   ?person rdf:type foaf:Person;
       foaf:name ?name
           optional{?person scinet:alias ?alias.}}


Prefixes like “foaf” indicate to which sub-ontology the concept or relation belongs. The portion in braces represents patterns for triples that the query will attempt to match against triples in the database. While the variables (terms prefixed with “?”) appearing after “select” are those desired to be returned, usually in tabular form. A graph can be constructed from the results. A set of triples (i.e. a graph) can be obtained directly by using the keyword “construct” instead of “select” in a somewhat different format. In either case, the graph would comprise a number of isolated subgraphs whose center is the ID of a person and whose edges connect the ID to either a preferred name or an alias. This illustrates the power of ontology-based network representations: A few standard queries are all that is required to extract a network from heterogeneous linked data.

### Using Database Queries to Construct Networks

The possible forms of the triples in the Sci-Net database can be inferred from Figure 2 by connecting any of the concepts there through the relationships shown. For this paper, we were mainly interested in how a series of workshops might bring together researchers from various countries and organizations, and how those relationships might change over time. With this in mind, we chose the following main entities:

- scinet:Meeting
- foaf:Person
- org:Organization
- foaf:Group
- scinet:Country
- foaf:Document

where the prefixes indicate the ontologies from which these concepts were drawn. Using predicates from the ontologies, the triples (relationships) of principal interest were:

- (Document, dc:creator, Person)—i.e., publications and their authors,
- (Person, org:memberOf, Group)—personal affiliations with research groups,
- (Person, org:memberOf, Organization)—organization affiliations,
- (Person, scinet:hasLocation, Country)—country of residence, and
- (Person, scinet:contributorTo, Meeting)—meeting attendees.

These specific entities and relationships were chosen to enable us to build networks of people annotated by group, organization, and country affiliation, and linking them to the meetings they attended. Focusing on a series of meetings, specifically the sequence of workshops sponsored by Los Alamos National Laboratory (LANL) over a period of several years, provided the opportunity to see how the networks evolved over time. In this context, the main relationship for inferring interpersonal connectivity was meeting attendance, i.e., two persons were assumed to be connected if they attended the same meeting.

To illustrate the utility of an ontology-based approach, the following example shows how we extracted, from a much larger set of persons, only those who attended a sequencing workshop:


  select distinct ?person ?personName
?workshop ?workshopName ?country
?countryName ?region ?regionName
      where {
      ?workshop rdf:type scinet:Workshop;
    rdfs:label ?workshopName.
  filter(regex(?workshopName, “sequencing”))
  ?person rdf:type foaf:Person;
      foaf:name ?personName;
      scinet:contributorTo ?workshop;
      scinet:hasLocation ?country.
  ?country a scinet:Country;
      foaf:name ?countryName.
  ?region a scinet:Region;
      foaf:name ?regionName.
  ?region apro:contains ?country;
      apro:containedIn scinet:who_regions.
}


This query returns the entire graph of meeting participants, the workshops they attended, resident country, and in this case, the WHO region of the country.

Rather than form more complex queries to build related subgraphs, we imported this graph into Python programs and used the NetworkX graph analysis package for further analysis (Hagberg et al., 2008). This allowed us more control in manipulating the graph and provided several standard output formats for visualization.

## Results

Our analysis focuses on the impact of a series of international genomics and sequencing training workshops for biosurveillance conducted by LANL. BTRP has supported participation in these workshops by country partner scientists and laboratorians since 2012. Each year, the training includes experience in both laboratory-based sequencing and bioinformatics for pathogen detection, and participants choose which section they attend. This annual course runs for 1 week with a typical participation of 25 researchers from 10+ countries. Since 2013, a total of 180 participants (126 unique) from 21 countries have taken part in these events. They are generally scheduled to coincide with a Sequencing, Finishing, and Analysis for the Future scientific conference. These workshops fill a crucial training need. Reduction in the costs of genomic sequencing and the creation of bioinformatic analysis methods has led to a democratization of sequencing technologies for application to biosurveillance. While detection of microbial pathogens has been accomplished with PCR assays, new advances in sequencing technologies can now be used to investigate whole genomes of microbes, characterize complete microbiomes, and determine co-infected hosts. Furthermore, phylogenetic relationships, transcriptomics, and gene function can be characterized for individual pathogens. These rapid breakthroughs in genomics applied to biosurveillance require advanced training in sequencing laboratory techniques and bioinformatics. Post-graduate degree programs in infectious diseases and microbiology only a few years ago may not have offered sequencing opportunities or experience.

### The Sequencing Workshop Network

**Figure 3:**
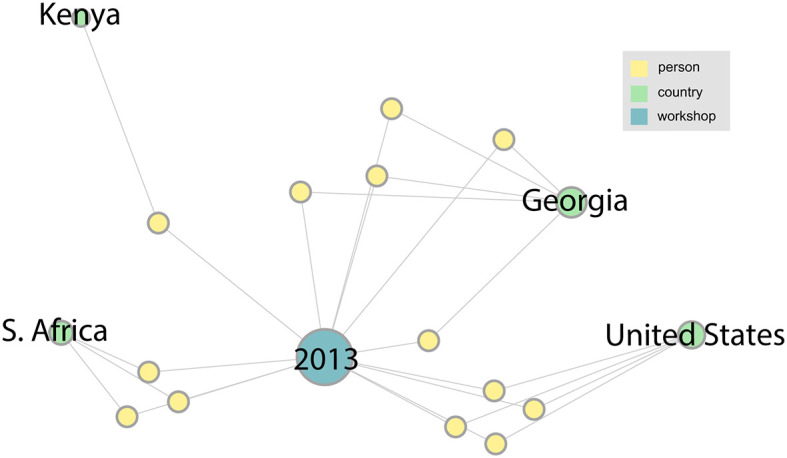
The network associated with the LANL 2013 sequencing workshop. In this figure and the ones to follow, information about persons (yellow nodes) has been anonymized by leaving them unlabeled.

We describe the networks that were derived from our analysis of the LANL sequencing workshops from 2013 to 2019. Summary statistics for this data are shown in Table 1. The following figures (Figure 3 through **Figure 6**) are a series of multidimensional, multipartite graphs showing the cumulative growth of the network between 2013 and 2019. Each figure builds on the graph depicted in the previous figure, adding new nodes and edges to capture the additional entities and relationships that were added to the network since the previous time step. These graphs were produced using Gephi graph visualization software (Bastian et al., 2009). The full 2019 graph was laid out using a force-directed graph layout algorithm; earlier versions of the graph were then generated by filtering out the nodes and edges from later years, while retaining the same layout as the 2019 graph for visual consistency. The size of the nodes (persons, countries, and workshops) in each of these figures indicates their degree (i.e., the total number of edges connected to the node). We have left only the person nodes unlabeled in these figures to simplify labeling and preserve anonymity. The network from the initial 2013 workshop is shown in Figure 3. Since this is the first in the series, it is a small network with 13 people and only four countries represented: South Africa, Kenya, Georgia, and the United States.

**Table 1:** Summary statistics for all workshops (2013–2019).

**Data type**	**Number**
Workshops	7
Participants	126
Countries	21
Organizations	58
BTRP Groups	2
Documents	137

**Figure 4:**
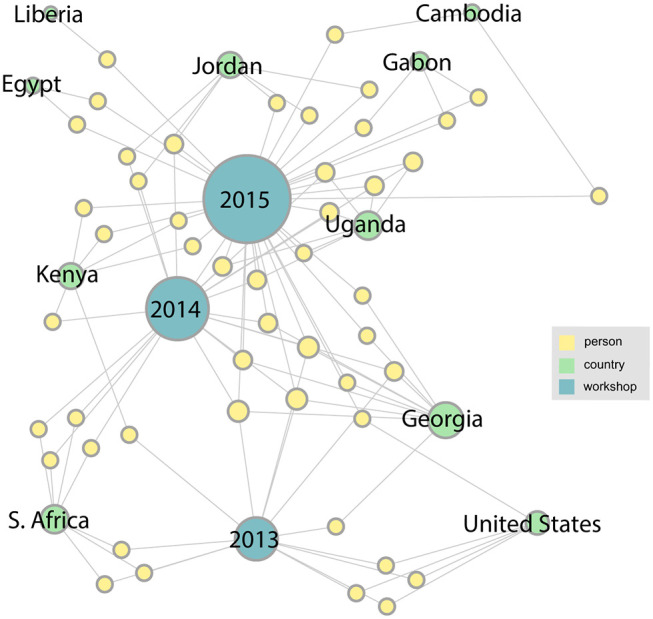
By 2015, with the addition of two more workshops in the series, the network becomes more complex, with several new participating countries appearing.

With the addition of links from two more workshops in the series, the network becomes more complex and takes on more structure. Figure 4 shows how the network has grown by 2015. We can see that the number of participating countries has grown considerably and many more organizations are represented. A major research collaboration group, the Western Asia Bat Research Network (WAB-Net), was established, bringing a number of new participants to the workshop, mainly from Georgia. There also appear to be several people who have attended multiple workshops in the series.

**Figure 5:**
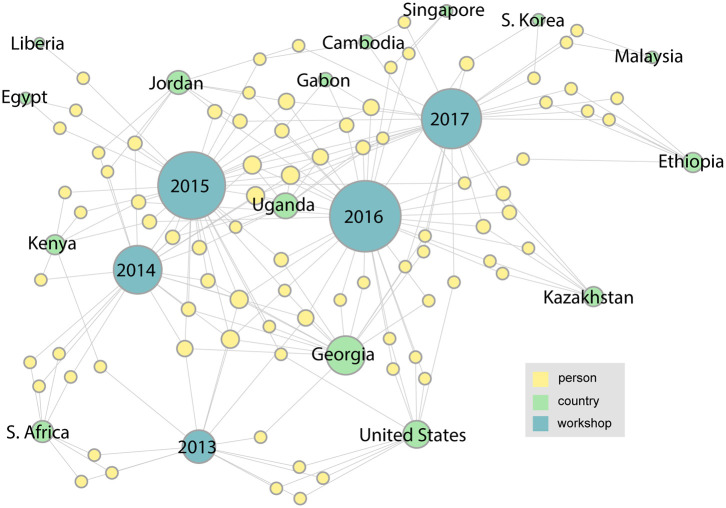
In the 2017 network it is evident that the number of participants and the diversity of countries and organizations has continued to grow.

**Figure 6:**
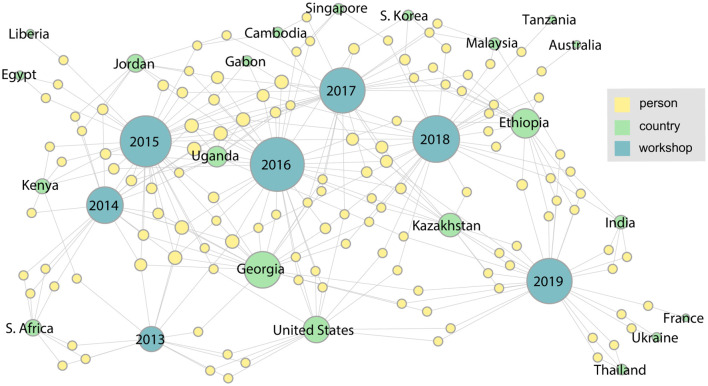
The network for the entire series of workshops from 2013 through 2019.

Two more workshops were completed by 2017, and as Figure 5 shows, the number and diversity of participating countries and organizations has increased. This trend continues through 2019 as shown in the final network (Figure 6). This figure shows the complete network encompassing all seven workshops, which includes 21 countries and 126 participants. From this final network, Georgia emerges as the country with the highest degree. These figures can also help identify countries that have not been as well-represented since their initial meeting, such as Liberia, Egypt, and Tanzania.

**Figure 7:**
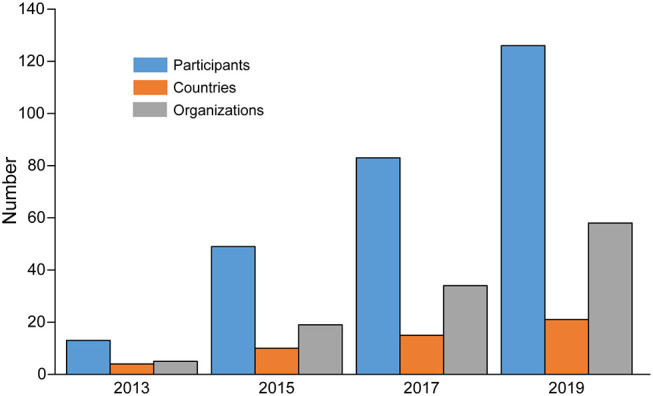
Growth of the network over time, showing the cumulative number of participants, countries, and organizations from 2013 to 2019.

The change in participation in the research networks over time is shown in Figure 7. Here we have plotted the number of participants, countries, and organizations over the period from 2013 to 2019. We see an increase in each category over time, with the number of participants and organizations seeing the greatest growth rates, while the number of countries grows at a slower rate.

### The Workshop Co-attendance Network

The multidimensional network visualizations presented above are one way of getting a sense of how the workshops have brought people together. It is clear from these how a diverse set of countries and organizations became interlinked as the number of participants grew over the time. It is also of interest to see and analyze the network of interactions between individual participants. Based on our dataset, there are various ways that participants can be linked to each other indirectly, via their shared connections to other entities. To capture the influence of the workshops, we chose to focus on the network defined by co-attendance at workshops, i.e., creating a person-person link whenever two individuals both attended the workshop in a given year. Since even the larger workshops were comparatively small, we believe networks derived from co-attendance should be reasonable indicators of potential interaction.

**Figure 8:**
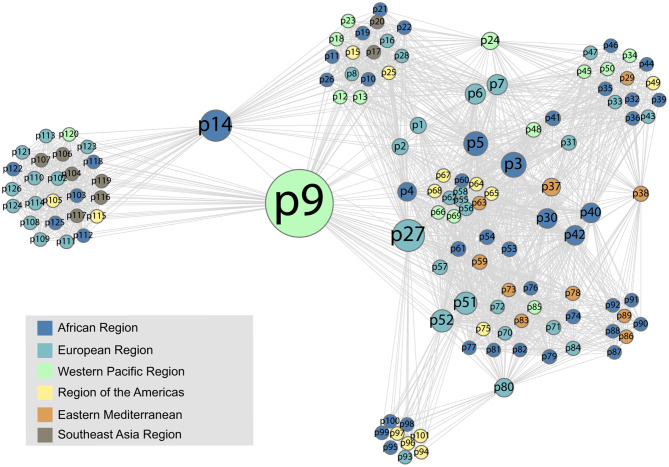
The social network formed by participants who attended the same meeting. The colors represent the regional affiliation of the attendees. We use anonymized, numerical participant labels here to facilitate comparison with Figure 9.

**Figure 9:**
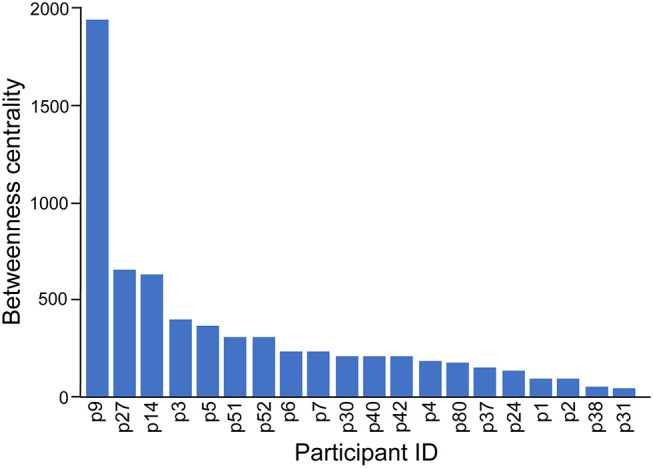
The top 20 participants by betweenness centrality in the social network of workshop participation.

The co-attendance social network is shown in Figure 8, again visualized with a force-directed layout algorithm using the Gephi software package. The nodes represent individual participants, and their colors show in which region their organization is located. Nodes are scaled by their betweenness centrality, answering the question of how likely one is to encounter that node while traversing an arbitrary path from one node to another in the network. Figure 9 shows the betweenness statistics for the 20 participants with highest betweenness centrality scores. Of these 20 participants, 9 are from the European Region and 7 are from the African Region, suggesting that participants from these countries play a particularly important role in connecting the community. More specifically, Figures 8, 9 suggest there are a relatively small number of participants (especially p9, p14, and p27) whose betweenness centrality is high compared to others. These individuals, who all attended more than one meeting, are key bridges that bring the larger network together. They have the potential to serve as conduits of information across the network, possibly putting them in a position of influence within the community. Having a limited group of people attend multiple meetings, giving them high betweenness centrality, is an efficient way of achieving network-wide connectivity while continuing to expand and diversify meeting participation.

It is also interesting to see that two tightly connected sub-networks seem to comprise the network as a whole (Figure 8). The smaller cluster on the left side of the graph corresponds to the most recent (2019) workshop, which is connected to the large network by only two individuals, p9 and p14, who had participated in previous workshops. The larger cluster on the right of the figure encompasses all of the previous workshops, which appear to have had more participants in common. This means that p9 and p14, who are from Cambodia and Ethiopia, respectively, play a key role as connections between the 2019 workshop participants and the rest of the network. This structure indicates that the 2019 workshop attracted an unusually high number of new participants compared to previous workshops, which have more overlap with each other in terms of attendance.

More cumulative network statistics are shown in Table 2. Unsurprisingly, these show a general increase in the number of links between participants as the network grows; by 2019, a person who participated in at least one of these workshops over the years made a connection to an average of 34 other participants, with a final network diameter (the longest distance between any two participants) of 3, both potentially useful metrics for the community-building success of the workshops.

**Table 2:** Cumulative network statistics for the workshop co-attendance network from 2013 to 2019.

**Year**	**Total links**	**Mean degree**	**Network diameter**
2013	78	12.0	1
2015	692	28.3	2
2017	1,435	34.6	3
2019	2,118	33.6	3

*Number of links corresponds to the number of cases where two individuals attended at least one workshop together. Mean degree is the mean number of people participants were connected to by attending at least one workshop together. Network diameter is the longest distance between participants based on co-attendance of workshops*.

### Coauthorship Networks Among Workshop Participants

**Figure 10:**
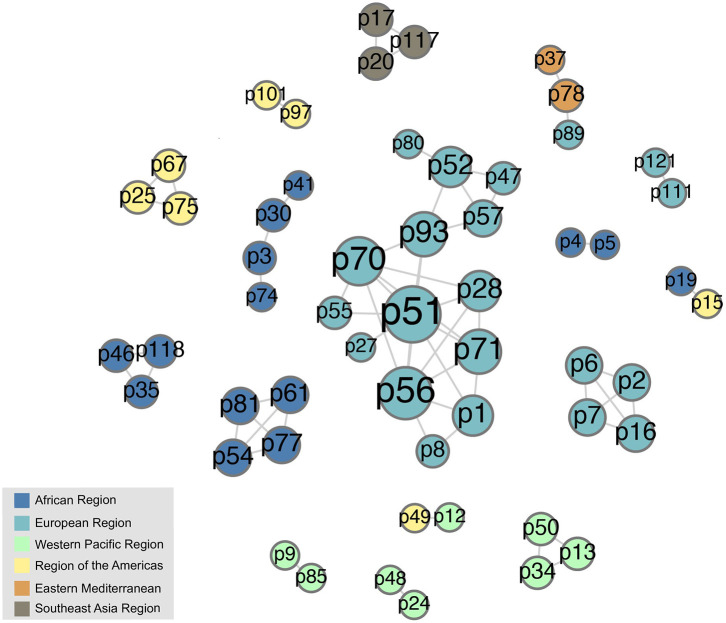
Network of 2013–2019 workshop attendees who coauthored publications with other attendees during 2014–2019. Note that this graph includes all workshop participants in this category as of 2019, so some links may reflect papers that were authored before the individuals in question actually attended a workshop. Nodes are sized by degree (i.e., number of publication collaborators).

In order to understand how workshop participation was related to other forms of collaboration and networking, we conducted a bibliometric analysis of coauthorship on journal publications among workshop participants. To do this, we looked only at the subset of participants who had coauthored a paper with at least one other workshop participant between 2014 and 2019. The complete graph of these coauthorship relationships is shown in Figure 10. This figure was drawn in Gephi using a force-directed layout, with node size indicating degree; links between participants are unweighted, i.e., they do not reflect the number of times participants were coauthors. Although it is not possible to determine whether these coauthorship relationships were driven by workshop participation, this does suggest that many workshop participants are also connected in other ways within the larger research community. However, we note that most of the collaboration communities shown in Figure 10 are made up of participants from a single region, and in fact many of them are made up of participants from a single country. This indicates that the workshops have not yet had a major impact on international collaboration on publications, even if they may have fostered other forms of international cooperation.

Statistics for the coauthorship network over time (Table 3) provide some additional insight. Here, it is important to note that the coauthorship network for each year is drawn from the total population of workshop participants from all years, 2013 to 2019, who coauthored papers together. That is, if two people who attended for the first time in 2019 wrote a paper together in 2014, this would be included in the network. The advantage of this approach is that, by observing the same group of attendees over a period of several years, we are able to show the growth in collaboration within this fixed-size group over time. As shown in Table 3, the number of workshop participants with coauthorship relationships increased from 19 to 55 from 2014 to 2019, and this group had jointly contributed to 137 papers by 2019. The number of network components linked by coauthorship relationships increased from 7 in 2014 to 16 in 2019, with the maximum component size growing from 6 to 14 over the same period. Although it is not surprising that the number of collaborations within a group might grow to some extent over time, these statistics indicate that there is an active community of collaborators among workshop participants who have continued to work together over time. However, we do not know whether these collaboration patterns were caused by workshop attendance or may be related to workshop participation in some other way.

**Table 3:** Network statistics for workshop participants' publication coauthorship network, 2014–2019.

**Year**	**Total authors**	**Total publications**	**Mean degree**	**Number of connected components**	**Mean component size**	**Maximum component size**
2014	19	10	2.00	7	2.71	6
2015	29	29	1.79	11	2.64	7
2016	44	60	1.86	15	2.93	9
2017	47	92	2.17	15	3.13	10
2018	48	115	2.25	14	3.43	14
2019	55	137	2.29	16	3.44	14

*2019 statistics describe the graph shown in Figure 10. The data may include papers that were authored before the individuals in question actually attended a workshop. Total authors is the number of workshop participants who collaborated with other workshop participants on publications and is cumulative over time. Total publications is the total number of publications with two or more workshop participants as coauthors and is cumulative over time. Mean degree corresponds to the average number of collaborators per author. A connected component is a group of authors who are connected to each other through coauthorship links but disconnected from the rest of the participants. Mean component size is the average size of these connected groups. Maximum component size is the number of people in the largest connected group of collaborators*.

It is also notable that workshop participants who did coauthor papers with each other had much higher average betweenness centrality on the workshop attendance graph (Figures 8, 9) than those who did not (104.63 vs. 18.00). This provides another point of reference to suggest that people who attended workshops in multiple years may have more influence within the BTRP research community.

## Discussion

### Strengthening Threat Reduction Networks

International scientific collaboration is a diverse and complex social phenomenon. It encompasses different types of social, scientific, and professional networks spanning many countries, and involving many groups and organizations. These networks are engaged in many activities such as research projects, meetings, workshops, and other events, producing research products like journal articles, reports, and conference presentations on many different subjects. These products lead to further research, and are a strong incentive for forming new collaborative relationships, causing the community of researchers to grow.

In many cases, research networks can address challenges and missions of sponsors faster and with a greater return on investment than individual research teams (Fair and Fair, 2019). One goal of BTRP in support of TRNs and other scientific networks is to build collaborative networks of scientists that can depend on each other to sustainably address the continuing challenge of reducing the threat from dangerous infectious diseases. Preventing epidemics depends on detecting, diagnosing, and reporting on infectious diseases, which is better facilitated through networks of scientists and institutions working together. Through active collaboration and cooperation, the ultimate goal of global health security can be strengthened and sustained. As we write this paper, scientists within BTRP TRNs are playing a critical role in diagnosing COVID-19, understanding the virus, and assisting in the global response to this health crisis. The relationships formed through these networks have proven to be invaluable for sharing of information, samples, data, analyses, and support in working tirelessly to mitigate the pandemic.

In this paper, we have focused on the role of one small set of activities, a series of sequencing training workshops, in the development of research networks. Our analysis shows how this series of workshops, over a period of 7 years, continued to attract new and diverse participants, building an increasingly international network. At the same time, however, there were enough repeat attendees from year to year that the network as a whole remains connected across years. In particular, certain key people who attended more than one workshop show up as having high betweenness centrality, and play a key role in connecting the overall network. This structure was not planned by the workshop organizers, although it is perhaps not unusual for a series of professional meetings on a particular topic. However, this finding does suggest that if one of the goals of organizing workshops like these is to build a larger network of potential collaborators, organizers might want to deliberately plan for a mix of new and repeat attendees in order to reach the largest possible number of participants while maintaining links between events.

### Ontology-Based Analysis of Social Networks

The main goal of this paper was to demonstrate a method for storing, manipulating, and visualizing complex multidimensional research networks using an ontology-based graph database. Based on an example of a series of training workshops, we demonstrated how this method can be used to extract a subset of the larger multidimensional network to address a particular research or operational question, and showed how we could flexibly create different visualizations to illustrate features of interest. While this is a relatively straightforward example, we believe this overall approach has great potential for enabling more sophisticated studies of multidimensional networks. The SCINET ontology, specifically, provides a set of categories and relationships that could be applied or adapted to the analysis of a wide range of scientific research networks.

The use of ontologies in social network analysis is a powerful approach for exploring multiple types of possible links between multiple types of entities in a multidimensional network. Because ontologies capture meaning, it is possible to analyze relationships in collaborative research across a range of different contexts: e.g., by country or organizational affiliation, by research topic, and so on. Through use of database queries, the technology can be used to filter information and build relationships in multiple ways, depending on the research question and desired type of analysis. By capturing concept and relationship hierarchies, this approach also makes it possible to pose very general or very specific questions within the same framework. For example, we could look at the network created by events in general, or as we did in this paper, focus on workshops alone. Alternatively, we could have looked at collaboration networks around infectious diseases in general, or only diseases that are caused by bacteria, or only those that affect livestock. With a network like this, any set of entities or relationships that can be extracted by a database query creates a network to which the tools of network science may be applied. Even though our current analysis only scratches the surface of the potential of this approach, we believe this is a potentially powerful and flexible set of tools that could have widespread use in network analysis.

### Limitations and Future Work

As discussed previously, our data set, though broad and diverse in its coverage of BTRP research networks, was also uneven in its coverage of events and time periods. As a result, we were unable to address some research questions or make use of some of the more complex types of analysis that could be facilitated by our ontology-based approach. In addition, the diverse and non-standardized data sources we drew from required a significant manual coding effort to capture in database-ready triple format. This type of effort is not required in many bibliometric studies, where data is drawn from pre-existing bibliographic databases where entities like authors, titles, and affiliations are provided as distinct data fields, which facilitates automated data capture. To make our data ingestion process more scalable, we hope to implement more automated methods in the future. With this type of data, however, automation will likely require use of natural language processing tools that are capable of recognizing and categorizing named entities, rather than more standard bibliometric approaches. Other possible extensions of our work include use of more quantitative network science metrics and algorithms to characterize networks and development of user-oriented tools to enable non-specialists, such as program managers, to make use of our database to generate visualizations and reports that can help them understand, build, and sustain collaborative research networks.

## Data Availability Statement

The datasets generated for this study are available on request to the corresponding author.

## Ethics Statement

Ethical review and approval was not required for the study on human participants in accordance with the local legislation and institutional requirements. Written informed consent for participation was not required for this study in accordance with the national legislation and the institutional requirements.

## Author Contributions

JF and JA conceived of the overall idea. JA, WR, and MR developed the ontology and its database implementation. JA, BS, AB, and WR conducted analysis and produced visualizations. JA, BS, AB, and JF wrote the manuscript.

## Conflict of Interest

The authors declare that the research was conducted in the absence of any commercial or financial relationships that could be construed as a potential conflict of interest. The authors declare that this study received funding from Los Alamos National Security, LLC (2006–2018) and Triad National Security, LLC (2018-Present), operators of Los Alamos National Laboratory. The funder was not involved in the study design, collection, analysis, interpretation of data, the writing of this article or the decision to submit it for publication.
